# Transfer Printing of Perovskite Whispering Gallery Mode Laser Cavities by Thermal Release Tape

**DOI:** 10.1186/s11671-021-03646-4

**Published:** 2022-01-06

**Authors:** Guo-Hui Li, Bo-Lin Zhou, Zhen Hou, Yan-Fu Wei, Rong Wen, Ting Ji, Yi Wei, Yu-Ying Hao, Yan-Xia Cui

**Affiliations:** 1 College of Physics and Optoelectronics, Key Laboratory of Interface Science and Engineering in Advanced Materials, Key Lab of Advanced Transducers and Intelligent Control System of Ministry of Education, Taiyuan University of Technology, Taiyuan, 030024 China; 2 Key Laboratory of Materials Modification by Laser, Ion, and Electron Beams (Ministry of Education), Dalian University of Technology, Dalian, 116024 China

**Keywords:** Transfer printing, Perovskite nanocavities, Laser, Thermal release tape

## Abstract

The outstanding optoelectrical properties and high-quality factor of whispering gallery mode perovskite nanocavities make it attractive for applications in small lasers. However, efforts to make lasers with better performance have been hampered by the lack of efficient methods for the synthesis and transfer of perovskite nanocavities on desired substrate at quality required for applications. Here, we report transfer printing of perovskite nanocavities grown by chemical vapor deposition from mica substrate onto SiO_2_ substrate. Transferred perovskite nanocavity has an RMS roughness of ~ 1.2 nm and no thermal degradation in thermal release process. We further use femtosecond laser to excite a transferred perovskite nanocavity and measures its quality factor as high as 2580 and a lasing threshold of 27.89 μJ/cm^2^ which is almost unchanged as compared with pristine perovskite nanocavities. This method represents a significant step toward the realization of perovskite nanolasers with smaller sizes and better heat management as well as application in optoelectronic devices.

## Introduction

High-quality whispering gallery mode (WGM) perovskite nanocavities have attracted widespread interest for developing low threshold and narrow linewidth lasers due to their unprecedented optoelectronic properties, small sizes and low losses that are important for applications such as light sources for on-chip integration, high-performance lab-free sensing [1–6]. In order to realize high-quality WGM perovskite nanocavities, various methods have been developed including low-temperature solution processable synthesis, chemical vapor deposition methods (CVD) [7, 8]. Although high-quality perovskite nanocavities have been demonstrated by using low-temperature solution processable synthesis, the quality of the resulting WGM cavities is limited by the using of solvents, surfactants and other unintentional impurities [9, 10]. In contrast, CVD method with high level of controllability, versatility and scalability has been considered as a promising method for achieving high-quality WGM perovskite nanocavities [11]. Various high-quality WGM perovskite nanocavities have been successfully grown on mica substrate [8, 12, 13]. However, the difficulty in transferring WGM perovskite nanocavities to desired surfaces while maintaining high-quality limits their applications [14–16].

Methods including wet transfer using a polymer support, dry transfer using polydimethylsiloxane (PDMS) rubber or thermal release tape for transferring two-dimensional materials are not suitable for transferring WGM perovskite nanocavities. Wet transfer processes have been widely used for fabrication of high-quality devices using two-dimensional materials [17, 18]. However, this wet process is not suitable for the preparation of WGM perovskite nanocavities because the water exposing process will induce an irreversible degradation of the perovskite [19]. Dry transfer print solid objects from one substrate to another has been reported by using PDMS rubber as the elastomeric stamp [20, 21]. In order to peel off targets successfully, adhesion of the stamp should be strong enough to adhere the target to the stamp surface. During peeling process, change in available elastic and potential energy associated with a small contact area decrease is called energy release rate [22]. Therefore, the energy release rate for the tape-nanoplatelets interface should be higher than that for nanoplatelets-substrate interface. However, the energy release rate for PDMS stamp and nanoplatelets interface is smaller than its counterpart for nanoplatelets-substrate interface of CVD-synthesized materials [23, 24]. Hot transfer using thermal release tapes as temporary supports successfully overcame these drawbacks [25]. Hot-pressing has been demonstrated for efficient transfer of two-dimensional materials onto desired substrates [26, 27]. However, the hot transfer sometimes causes undesired thermal degradation on WGM perovskite nanocavities when the substrate was heated up at 398 K to release the cavities [28]. And the adhesion force should be precisely controlled to ensure the nanocavities can be peeled off without accompany any mica residuals that could lead to scattering loss [29].

In this work, we report an improved hot transfer method that uses hand press to press the tape on perovskite nanocavities to precisely control the energy release rate and hotplate to release the perovskite nanocavities to precisely control the adhesion and thermal degradation. The transfer method can peel off CVD-synthesized perovskite nanoplatelet on mica substrate and release them on target substrate maintaining high quality of WGM cavities for laser applications and avoiding mica residuals and thermal degradation, which is convinced by optical microscope images, atomic force microscope (AFM) analysis, and laser characterizations.

## Methods

**Fig. 1:**
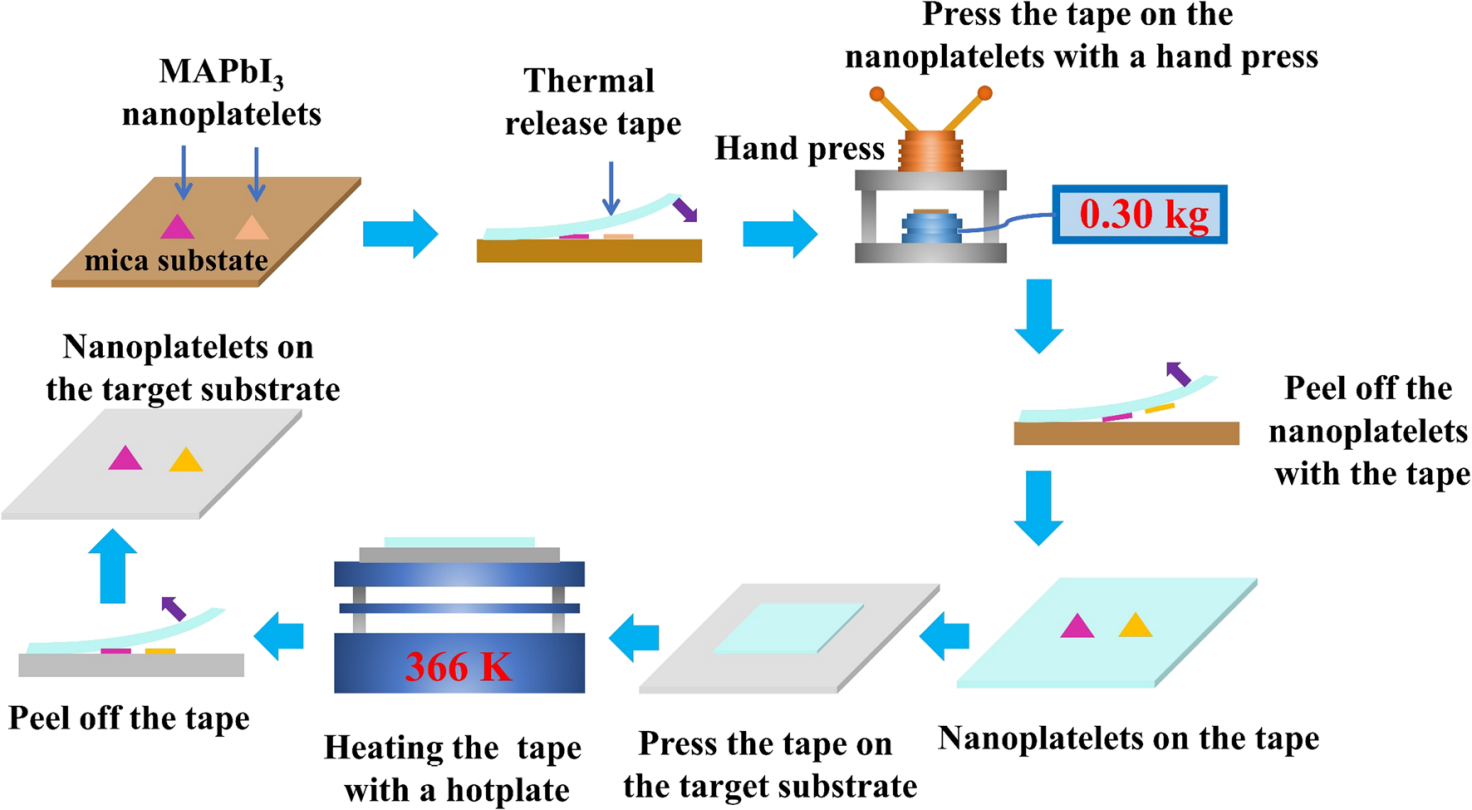
Schematic diagram of transfer printing process

Figure 1 shows the schematic diagram of the hot transfer method of CVD-synthesized perovskite nanoplatelet WGM cavities from a mica substrate to arbitrary substrates such as SiO_2_. The perovskite nanoplatelet WGM cavities have a thickness of ~ 100 nm and edge length of tens of micrometers. A commercially available thermal release tape in rectangular shape with 3-mm length and 2-mm width is used as the stamp. The perovskite nanoplatelet WGM cavities to be transferred is mechanical exfoliated with the thermal release tape. Nanoplatelets with thickness lower than 70 nm have a lower effective index which will cause a high radiative loss [30]. Thus, they cannot be used for lasing at room temperature. Nanoplatelets with thickness higher than 200 nm have higher absorption losses which break gain should be higher than losses condition of lasers. Thus, they cannot be used for lasing at room temperature either. The perovskite nanoplatelet WGM cavities are inspected under an optical microscope to select thinner (thickness of ~ 100 nm) nanoplatelets with regular shapes and edge lengths of 30–100 mm. Thanks to optical interference between light beams reflected from the top and bottom surfaces of the nanoplatelets, thinner nanoplatelet with difference thickness shows orange, pink colors under normal illumination. Atomic force microscopic image of MAPbI_3_ nanoplatelet shows that orange, pink, cyan nanoplatelets have thicknesses of ~ 100 nm [30].

Once perovskite nanoplatelet WGM cavities have been identified, the thermal release tape is pressed on the cavities. As the tape is transparent, we can see the nanoplatelet WGM cavities to be transferred through it and thus we can align the tape with the cavities precisely by using a microscope to optically locate the position of the nanoplatelet and a translation stage with metric micrometer to fine adjust the position of the nanoplatelet. In order to ensure the tape has appropriate adhesive forces that can exfoliate the cavities without new defects and mica residuals, the tape is pressed against the cavities by using hand press with precisely controlled pressure and pressing time. Cavities adhere to the surface of tape are mechanical exfoliated from the mica substrate with controlled separation speed (typically ~ 3 mm s^−1^). Since the adhesion between the cavities and the tape is rate-sensitive, pulling the tape away from the mica substrate with controlled speed led to adhesion that is strong enough to lift them away from the substrate without any mica residuals. The nanoplatelet cavities on the tape is attached to the large substrate and then transferred by using the hot-pressing processes.

In the hot-pressing processes, the tape/cavities/substrate is heated for 2 min in the hot-pressing process. To avoid thermal degradation of the perovskite nanoplatelet cavities, the heating temperature is ~ 366 K. After the transfer process, the tape is removed with a slower separation speed (~ 0.6 mm s^−1^) as the adhesive force does not disappear completely. Removing the tape with sufficiently slow separation speed can ensure the nanoplatelet cavities to adhere to the target substrate and separate from the tape.

## Result and Discussion

**Fig. 2:**
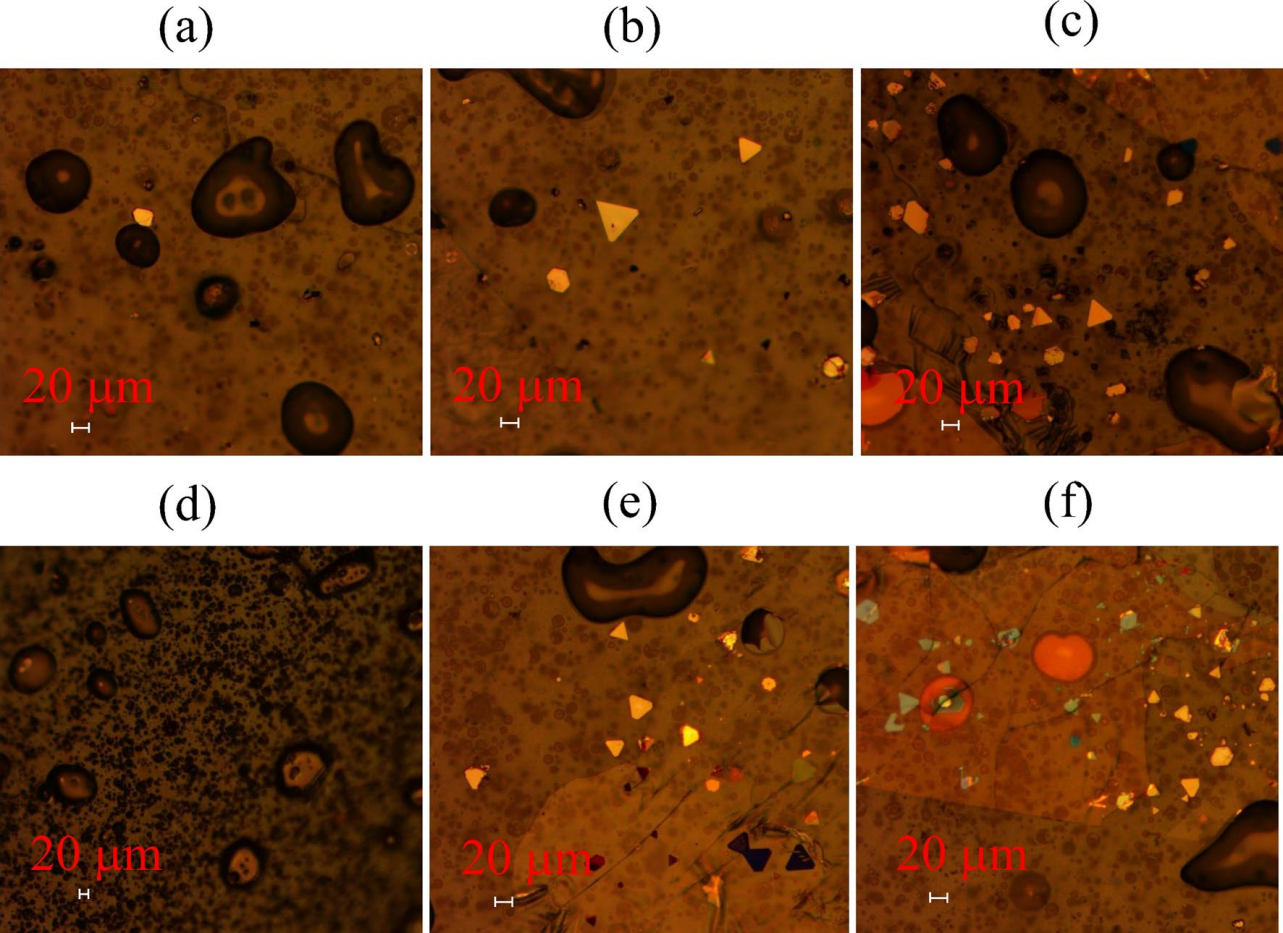
Microscopic images of perovskite nanoplatelets adhere to thermal release tape after pressing nanoplatelets/tape with 0.45 MPa (**a**), 0.50 MPa (**b**), 0.55 MPa (**c**) pressure for 3 min and 0.50 MPa pressure for 2 min (**d**), 3 min (**e**) and 4 min (**f**) before mechanical exfoliation

Figure 2a–c shows that the microscope images of the MAPbI_3_ nanoplatelets adhere to the thermal release tape obtained by mechanical exfoliating after pressing nanoplatelets with pressures of 0.45, 0.50, 0.55 MPa for 3 min, respectively. Under the pressure of 0.45 MPa, only one MAPbI_3_ nanoplatelet fragment is exfoliated. As shown in Fig. 2a, a yellow MAPbI_3_ nanoplatelet fragment with length longer than 20 mm adheres to the tape. When the pressure is increased to 0.50 MPa, more than three undamaged MAPbI_3_ nanoplatelets adhere to the thermal release tape after the mechanical exfoliation as shown in Fig. 2b. As the pressure increased to 0.55 MPa, a growing number of MAPbI_3_ nanoplatelets adhere to the thermal release tape after the mechanical exfoliation as shown in Fig. 2c. Unfortunately, the surface of MAPbI_3_ nanoplatelets are covered with mica sheets. And the number of damaged nanoplatelets increased due to the large pressure. Therefore, pressing nanoplatelets/tape with a pressure of 0.50 MPa can exfoliate the nanoplatelets without damage and residual mica.

Figure 2d–f shows that the micrograph of MAPbI_3_ nanoplatelets adhere to the heat-release tape exfoliated by pressing the nanoplate/tape with a pressure of 0.50 MPa and pressing time of 2, 3 and 4 min, respectively. As shown in Fig. 2d, few MAPbI_3_ nanoplatelets adhere to thermal release tape when mechanical exfoliated after pressing nanoplatelets/tape for 2 min. As the nanoplatelets/tape pressing time increased to 3 min, more than four regular shape MAPbI_3_ nanoplatelets were adhered to the thermal release tape after mechanical exfoliation. However, as the nanoplatelets/tape pressing time increased to 4 min, not only MAPbI_3_ nanoplatelets but also mica fragments adhere to the thermal release tape after mechanical exfoliation, and more nanoplatelets were broken during the mechanical exfoliation. It can be seen that the adhesion can be precisely controlled by the pressing time of the nanoplatelets/tape. The adhesion increases with the growing pressing time. Therefore, appropriate adhesion that can mechanical exfoliates nanoplatelets without damage and mica fragments can be realized by controlling the pressure and the pressing time. Our studies show that pressing the nanoplatelets/tape with a pressure of 0.5 MPa and a pressing time of 3 min before mechanical exfoliation can provide an appropriate adhesion that can transfer nanoplatelets to the thermal release tape without damage and mica fragments.

**Fig. 3:**
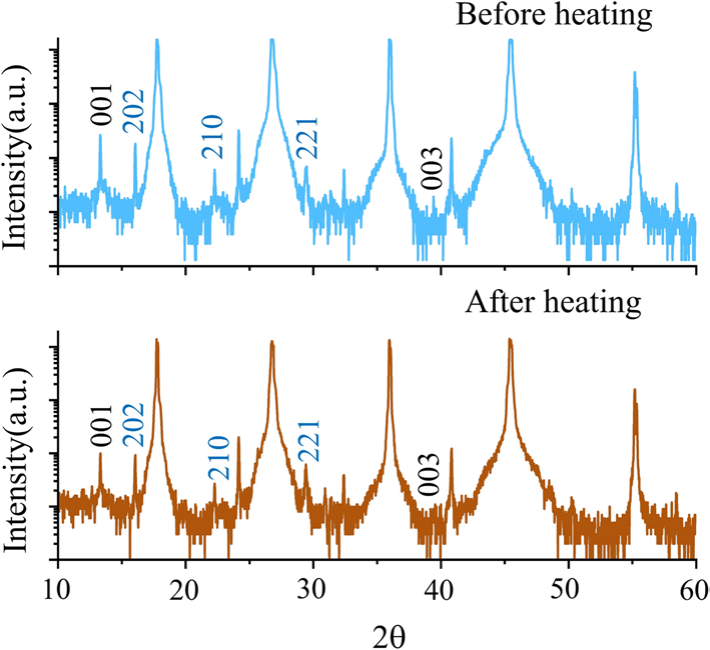
XRD pattern of pristine MAPbI_3_ nanoplatelets on mica substrate before (upper panel) and after (lower panel) thermal effect

In situ TEM observation of the thermal response of perovskite-base solar cell shows that perovskite layer can be stable for short times until 423 K. In order to avoid heat induced degradation of the perovskite during the thermal release process, we investigated the thermal stability of the perovskite nanoplatelets. During the experiment, the thermal stability of the perovskite nanoplatelets was tested with continuous heating at 367 K for 2 min. We monitored the XRD pattern of the perovskite nanoplatelets before heating and after heating. Figure 3 shows a detailed XRD pattern of the pristine perovskite nanoplatelets. Before heating, the specimen exhibits tetragonal perovskite crystal structure with small (202), (210), and (221) peaks and also small (100) and (003) peaks of PbI_2_ as shown in upper panel in Fig. 3. Then, the specimen is heated at 367 K for 2 min in ambient air condition. The resulting XRD pattern (lower panel in Fig. 3) reveals that the relative intensity of the tetragonal perovskite crystal structure peaks and PbI_2_ peaks almost unchanged. The MAPbI_3_ does not degrade to PbI_2_ during the heating process in our experiment. Therefore, the CVD-synthesized perovskite nanoplatelet is stable under moderate heating at 367 K for short time (~ 2 min).

**Fig. 4:**
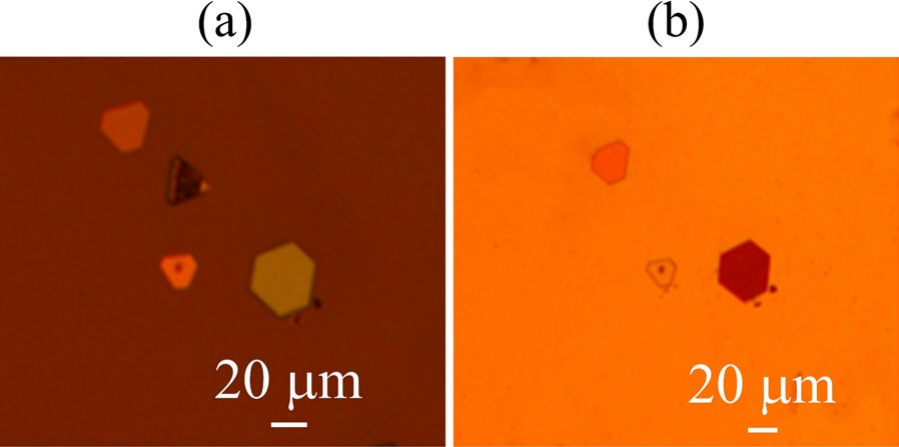
Microscopic images of pristine MAPbI_3_ nanoplatelets (**a**) and transfer printed MAPbI_3_ nanoplatelets (**b**)

Thermal release tapes with undamaged MAPbI_3_ nanoplatelets were pressed on the target substrate gently to ensure they are in full contact. After heating nanoplatelets/tape at 366 K for 2 min with a hotplate, the thermal release tape was removed at a sufficiently low separation speed of 0.6 mm/s to ensure the nanoplatelet adhere preferentially to the target substrate and separate from the thermal release tape. As can be seen, the nanoplatelet on mica substrate (Fig. 4a) are transferred to SiO_2_ substrate (Fig. 4b) without any damage.

**Fig. 5:**
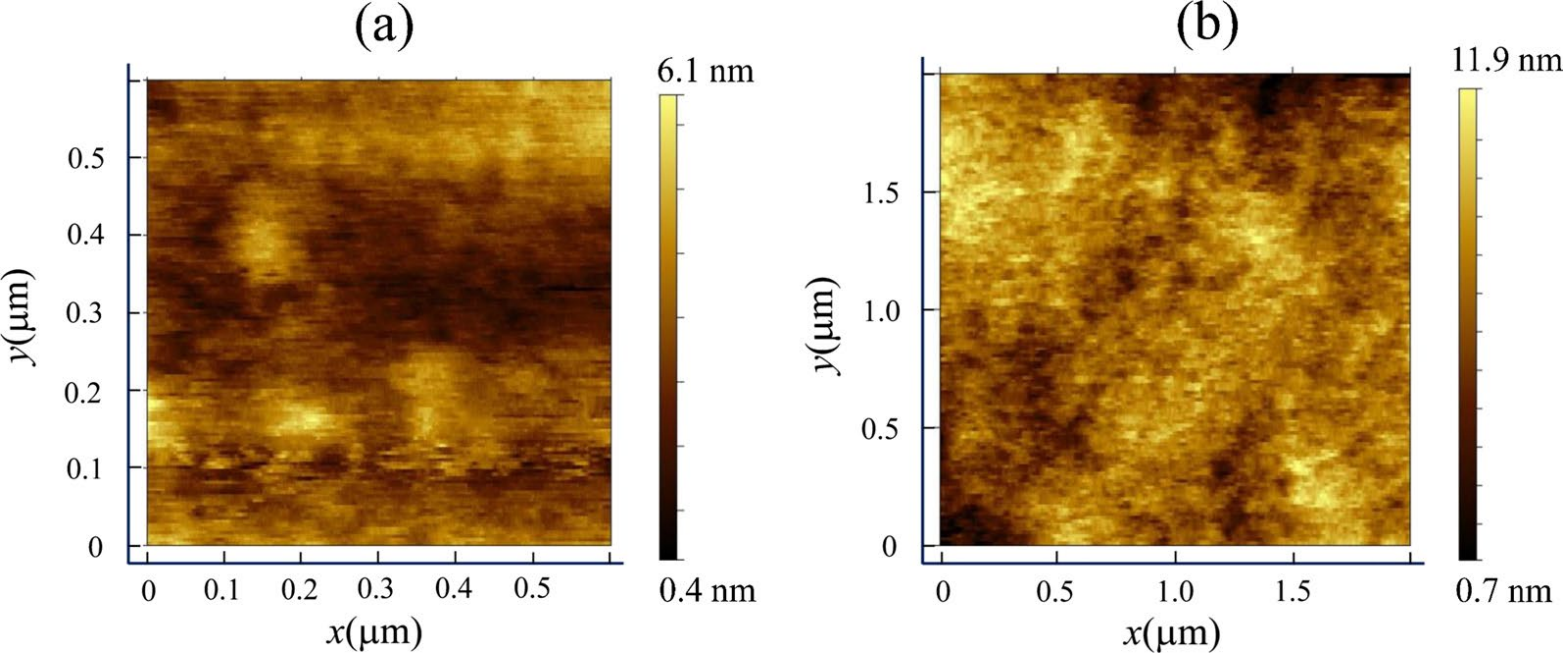
**a** AFM image of pristine MAPbI_3_ nanoplatelet showing RMS roughness of ~ 0.96 nm, **b** AFM image of transfer printed MAPbI_3_ nanoplatelet showing RMS roughness of ~ 1.18 nm

We also characterized the surface morphology of nanoplatelet on mica substrate and on transferred nanoplatelet on SiO_2_ substrate by atomic force microscope. Figure 5a shows a detailed surface morphology of the pristine perovskite nanoplatelets. As can be seen, the pristine perovskite nanoplatelet has a smooth surface with an RMS roughness less than 0.96 nm. After transfer printing, the surface morphology of transferred nanoplatelet on SiO_2_ substrate is well-preserved with an RMS roughness less than 1.18 nm as shown in Fig. 5b. The slightly higher roughness is caused by increased scanning range of the transferred nanoplatelet on SiO_2_ substrate.

**Fig. 6:**
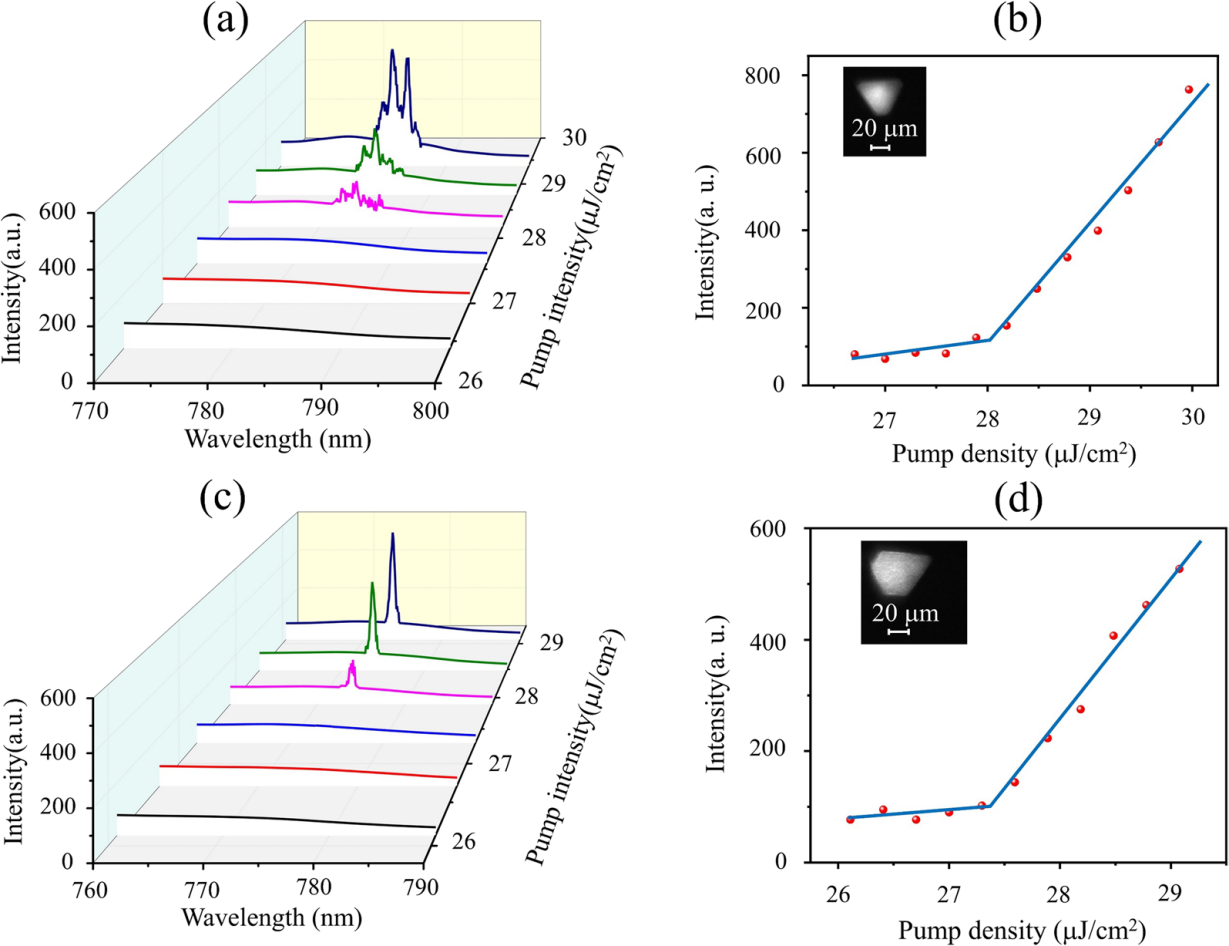
**a** Evolution of emission spectra obtained at different pump densities using pristine perovskite nanoplatelet on mica substrate. **b** Laser output intensity as a function of pump density using pristine perovskite nanoplatelet on mica substrate. **c** Evolution of emission spectra obtained at different pump densities using transfer printed perovskite nanoplatelet on SiO_2_ substrate. **d** Laser output intensity as a function of pump density using transfer printed perovskite nanoplatelet SiO_2_ substrate

Pristine perovskite nanoplatelet on mica substrate before transfer printing and perovskite nanoplatelets on SiO_2_ substrate after transfer printing were optically pumped at room temperature by a femtosecond-pulsed laser through a home-built fluorescence microscope. For comparison, we select a pristine perovskite nanoplatelet on mica substrate. As shown in Fig. 6a, at low pump density *P* (< 28.19 μJ cm^−2^), each emission spectrum shows a broad peak centered at ~ 770 nm with a full-width at half-maximum (FWHM) of Δ*λ* = 48 nm, which corresponds to spontaneous emission (SPE). At higher pump density *P*(> 28.19 μJ cm^−2^), the emission spectrum changes from a broad spectrum to a narrow spectrum which is one of the key properties of lasing. At *P*_Th_ = 28.78 μJ cm^−2^, a sharp peak at 783.67 nm appears and grows rapidly with increasing *P*, and the intensity of the broad SPE peak (non-lasing) remains almost constant. The FWHM at *P* = 28.78 μJ cm^−2^, at which pump density the lasing peak dominates, is 0.5 nm. It indicates that the laser has a FWHM linewidth more than an order of magnitude narrower than the typical linewidth (~ 10 nm) of ASE in an organic semiconductor [31, 32]. The light-in-light-out curve in Fig. 6b shows a slow increase in emission intensity with increasing pump density below the pump density of ~ 28.19 μJ/cm^2^, and then a faster increase in emission intensity thereafter. The nonlinear dependence of the emission intensity on the pump intensity is another the key properties of lasing. Therefore, the transfer printed perovskite nanoplatelet laser have a narrow linewidth of 0.5 nm and a clear threshold at 28.19 μJ cm^−2^.

Lasing performances of a perovskite nanoplatelet on SiO_2_ substrate after transfer printing was also tested. As shown in Fig. 6c, at low pump density *P* (< 27.59 μJ cm^−2^), each emission spectrum shows a broad peak centered at ~ 770 nm with a full-width at half-maximum (FWHM) of Δ*λ* = 48 nm, which corresponds to spontaneous emission (SPE). At higher pump density *P*(> 27.59 μJ cm^−2^), the emission spectrum changes from a broad spectrum to a narrow spectrum which is one of the key properties of lasing. At *P*_Th_ = 27.89 μJ cm^−2^, a sharp peak at 774.06 nm appears and grows rapidly with increasing *P*, and the intensity of the broad SPE peak (non-lasing) remains almost constant. The FWHM at *P* = 27.89 μJ cm^−2^, at which pump density the lasing peak dominates, is 0.3 nm. It indicates that the laser has a FWHM linewidth more than an order of magnitude narrower than the typical linewidth (10 nm) of ASE in an organic semiconductor. The light-in-light-out curve in Fig. 6d shows a slow increase in emission intensity with increasing pump density below the pump density of ~ 27.89 μJ/cm^2^, and then a faster increase in emission intensity thereafter. The nonlinear dependence of the emission intensity on the pump intensity is another the key properties of lasing. Therefore, the transfer printed perovskite nanoplatelet laser have a narrow linewidth of 0.3 nm and a clear threshold at 27.89 μJ cm^−2^. It can be seen that the transfer printed perovskite nanoplatelet on SiO_2_ substrate shows almost similar lasing performances as the pristine perovskite nanoplatelets on mica substrate. It indicates that the transfer printing of perovskite nanoplatelets by thermal release tape can provide an efficient method to combine the perovskite nanoplatelets with various substrate for laser applications.

## Conclusion

In summary, we have developed and demonstrated a method for transfer printing of perovskite whispering gallery mode nanoplatelets laser using thermal release tape. Under optimal conditions, the CVD grown perovskite nanoplatelets can be efficiently transferred and are crack-, wrinkle-, and residual glue free. The high-quality transfer not only preserves the structural integrity, but also the optical properties of the whispering gallery mode cavities, which is critical for developing new perovskite whispering gallery mode lasers such as plasmonic lasers and high heat dissipation lasers. Transfer printing of perovskite nanoplatelets can also be generalized to transfer printing of other perovskite whispering gallery mode cavities.

## Data Availability

The experiment data supporting the conclusion of this manuscript have been given in this manuscript. All data are fully available without restriction.

## Abbreviations

WGM: Whispering gallery mode
CVD: Chemical vapor deposition methods
PDMS: Polydimethylsiloxane
AFM: Atomic force microscope
FWHM: Full-width at half-maximum
SPE: Spontaneous emission
